# Editorial: The tug of war between parasites survival and host immunity

**DOI:** 10.3389/fimmu.2023.1234191

**Published:** 2023-06-19

**Authors:** Rahul Shivahare, Swati Dubey, Bradford S. McGwire

**Affiliations:** ^1^ Division of Infectious Diseases, Department of Medicine, Wexner Medical Center, The Ohio State University, Columbus, OH, United States; ^2^ Department of Pathology and Laboratory Medicine, University of California Los Angeles, Los Angeles, CA, United States

**Keywords:** host-parasite interactions, protozoan parasites, helminth parasites, host defense, pathogenesis, innate immunity

Parasitic diseases cause significant harm in tropical and sub-tropical countries, leading to mortality, morbidity, and socio-economic disparities. The outcome of the infection and the severity of the disease often depend on the interplay between parasite survival strategies and the host immune response. A strong and effective host immune response can limit parasite replication, reduce disease severity, and promote recovery. Conversely, if the parasite can evade or suppress the immune response, the infection can persist and lead to chronic or severe disease. Sometimes the host immune response itself can contribute to disease pathology through excessive or dysregulated immune reactions triggered by parasitic infections, leading to tissue damage, inflammation, and immune-mediated disorders.

This tug of war between parasites and host immunity is driven by coevolutionary dynamics. Parasites continuously adapt to host immune responses, and hosts, in turn, develop new strategies to counteract parasite evasion tactics. This coevolutionary process can lead to the emergence of new parasite strains with enhanced immune evasion abilities (thus fostering drug resistance) and host genetic variations that confer resistance or susceptibility to specific parasites. Thus, understanding the intricate mechanisms and dynamics involved in this ongoing battle is crucial for developing more effective approaches to manage and combat parasitic diseases. It involves studying the mechanisms employed by parasites to evade immune detection and developing interventions that can boost host immune responses. Additionally, exploring the genetic factors underlying host susceptibility to parasitic infections can help identify individuals at higher risk and inform targeted prevention or treatment approaches.

This Research Topic aims to contribute to our understanding of host–parasite interactions, leading to advancements in the field of parasitology and improvements in human and animal health. It comprises four original research articles and one review article focusing on trypanosomiasis, leishmaniasis, malaria, giardiasis (protozoan infections), and schistosomiasis (helminth infection).

The research paper by Nguyen et al. focuses on the infection caused by an extracellular protozoan parasite, *Trypanosoma evansi*, which leads to a lethal chronic wasting disease in livestock and game animals. This study has established an experimental disease model for chronic animal trypanosomiasis. The model demonstrates that the infection can persist for over 100 days, mimicking the key characteristics of chronic animal trypanosomiasis. The research employed a detailed single-cell RNA sequencing (scRNA-seq) analysis of spleen macrophages to understand the complex activation status of spleen macrophages, particularly tissue resident macrophages, and their role in regulating extramedullary erythropoiesis. The study highlights the disruption of erythroid differentiation and the consequent occurrence of chronic anemia, which contributes to the characteristic wasting syndrome observed in the late stages of the disease.

The review article by Chandley et al. highlights the importance of understanding host–parasite interactions and immune evasion mechanisms in the development of effective therapeutics against malaria. It emphasizes the need for research to fill the gaps in our knowledge and gain insights into these mechanisms for the development of vaccines and immunotherapies that can overcome immune evasion and induce long-lasting immunity against malaria. It also proposes the use of immunoinformatic approaches to identify novel vaccine antigens from different stages of the *Plasmodium* life cycle and develop multi-antigen or multi-stage vaccines.

The research conducted by Bouabid et al. aims to understand the mechanisms underlying the differences in disease progression observed in mouse strains infected with *Leishmania major*. The researchers analyzed dynamic transcriptome data obtained from bone marrow-derived macrophages (BMdMs) infected with *L. major* in mice strains that exhibited contrasting behaviors in response to the infection. By combining gene expression analysis with network propagation, the researchers were able to identify dynamically altered mouse strain-specific networks that provide mechanistic insights into the contrasting responses to the infection. This research contributes to a better understanding of the complex interactions between *Leishmania* parasites and host macrophages, potentially paving the way for future therapeutic interventions or preventive measures against leishmaniasis.

The study by Reinholdt et al. provides valuable insights into the complex dynamics of schistosomiasis and emphasizes the need to understand the interplay between the host immune response, parasite life cycle, and organ pathology for accurate diagnosis and management of the disease. This study investigates two aspects of schistosomiasis: the ability of unisexual worms to mate and the impact of the immune response on organ pathology. The researchers infected female mice with either male or female cercariae and examined the presence of worm mating and its effects on the host immune response and organ pathology. The results of the study show that single worms, even months after unisexual infection, were capable of mating and initiating oviposition. The deposition of eggs led to a typical Th2 immune response in the liver, characterized by increased recruitment of CD4+ T cells. However, the organ damage caused by the unisexual worms did not subside and served as a baseline for the emerging inflammation and fibrosis triggered by egg deposition. This highlights the importance of considering the infection status, even without positive egg detection, especially in areas with low prevalence of schistosomiasis.

The study by Yu et al. investigates how *Giardia duodenalis*, a parasitic protozoan, affects intestinal epithelial cells (IECs). They have found that *Giardia* infection leads to the activation of endoplasmic reticulum (ER) stress and unfolded protein response (UPR) pathways in IECs. The UPR signaling pathways, including IRE1, PERK, and ATF6, induce cell cycle arrest by upregulating p21 and p27 expression and promoting E2F1-RB complex formation. Additionally, UPR pathways (PERK and ATF6) promote apoptosis in infected IECs, while the IRE1 pathway has a suppressive effect on apoptosis. These findings provide insights into the mechanisms by which *Giardia* affects host cell responses and contributes to understanding the parasite’s pathogenesis.

Overall, these studies contribute to our understanding of host–parasite interactions and provide insights into the development of preventive and therapeutic interventions in parasitic diseases. We hope that this Research Topic has the potential to stimulate further research, innovation, and advancements in this field.

## Author contributions

All authors have contributed to the editorial and approved the final version.

